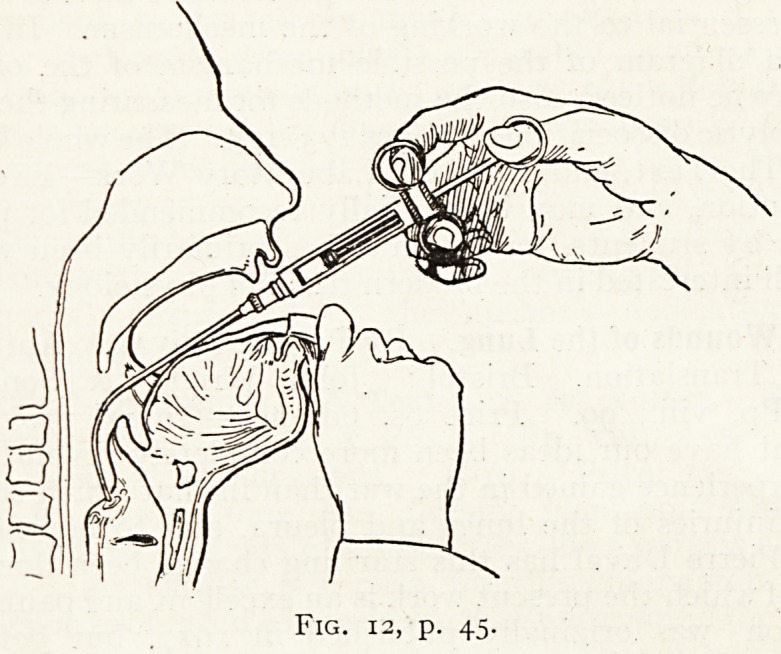# Book Review

**Published:** 1920-03

**Authors:** 


REFERENCES.

### Reviews of Books.

**Fevers in the Tropics.** By Sir LEONARD ROGERS, I.M.S. Third Edition. Pp. xii. 160. London: Oxford Medical Publications. 1919. Price 30s.—In these days, when so many of us have seen something of tropical diseases or their effects, the appearance of the third edition of this book is of especial interest. The best chapters are those on kala azar, enteric fever, amœbic hepatitis, malaria and the fevers of short duration, which are the diseases of which the author can speak from very large experience. Of those diseases rare or unknown in Bengal he gives able summaries of the present state of our knowledge. The clinical and therapeutic sections are those most fully given and are generally admirable; pathology, excepting the blood state, receives less attention. We are somewhat mystified by the references to the infection of European men with kala azar through their relation to the daughters of the soil. If it is a fact that there is known "no instance in which a European cohabited with an infected native woman and escaped kala azar," this is surely a very important epidemiological fact. The author does not suggest sexual intercourse as the cause of infection, but sees in it another fact to support the bed bug theory of kala azar, surely rather a strain on an unproved hypothesis. The paratyphoid fevers in India are treated somewhat superficially, and he even seems to doubt their specificity. We fancy if skilled bacteriological diagnosis was made available for the civil population in India the paratyphoid fevers would be proved to be commoner than the author imagines. As far as we can see from his method of diagnosis the paratyphoid infections would not be recognised. He recommends the agglutination test in a dilution of 1 in 100, and apparently to *B. typhosus* only. He gives the technique of blood culture, but does not give any results of this test in his own hands. If blood cultures were done in all early cases and stool and urine examinations in all later ones, and the blood serum put *up to titre to all three organisms* in non-inoculated patients, we should get more reliable results as to the incidence of the three types of infection. Some of the charts (*e.g.* those on p. 112) would do well for paratyphoid fever, and a Widal of 1 in 100 to *B. typhosus* might be a group reaction produced by a specific infection of Paratyphoid A. or B. The Diazo reaction, a very unreliable test, is given more prominence than it deserves, whereas Marris's atropine test is not referred to under enteric fevers, but is briefly mentioned under the diagnosis of infective jaundice (p. 168). "Infective jaundice, or *Spirochætosis icterohæmorragica*" is dealt with in some detail, but the author has made no clear distinction between it and the common camp jaundice. He refers to the Dardanelles outbreak as if it were probably spirochætal jaundice; but that epidemic was surely camp jaundice, similar to that which occurred in epidemic form in India and Mesopotamia, and was certainly not spirochætal jaundice in these two latter countries. We are told that infective jaundice (meaning presumably spirochætal jaundice) "has long been known to be widespread over the world, including India." We do not think this a correct statement for cases in which the presence of the spirochæte has been proved. The clinical picture of the two diseases is perfectly distinct, so is their pathology, but an inexperienced person reading this chapter would confuse the two. There is a short description of trench fever, though we doubt the justification for including this disease in a book on "Fever in the Tropics." The chapter on malaria is excellent and mirrors the author's great experience and the soundness of his conclusions. We note he has dropped the method he so strongly advocated of giving intravenous antimony injections in malignant tertian malaria, as he gives the barest reference to the method. He has also merged the "Seven day fever," which he formerly described as a separate entity, into dengue fever, and here he follows general opinion. The chapter on these short fevers is clear and informative, though, as we suggested before, there is a probability of mild attacks of paratyphoid fever being responsible for some of these obscure pyrexias. Despite these and other slight imperfections in the book, we can recommend it to the physician in tropical countries as cordially as we have former editions. It is a monument of the author's ability and indefatigable industry, and such defects as it has are the defects of its qualities.

**The "Nauheim" Treatment, in England, of Diseases of the Heart and Circulation.** By Leslie Thorne Thorne, M.D. Fifth Edition. Pp. vii., 160. London: Baillière, Tindall & Cox. 1918. Price 5s. net.—The Nauheim treatment as originally propounded consists of three parts, (1) graduated exercises, (2) baths, and (3) the Nauheim "atmosphere." The war has taught us the value of exercise as a prognostic test and as a therapeutic measure; at the same time it has cut us off from Nauheim and proved to us that the "atmosphere," *i.e.* optimism, can be cultivated anywhere by a physician who is determined to do so. What of the second? Perusal of Dr. Thorne Thorne's little book leaves us little room for doubt. The "Nauheim" baths do good, it is clear; whether more so than other and less expensive media of optimistic suggestion is not so clear. The whole thing works out to this, that the patient must be weaned from his fears, and so long as this is accomplished it does not much matter how. Dr. Thorne Thorne's book has reached a fifth edition, a fact that speaks for itself.

**The Early Diagnosis of Tubercle.** By Clive Riviere, M.D. Second Edition. Pp. xii., 314. London: Oxford Medical Publications. 1919. Price 10s. 6d. net.—The demand for a new edition of this work has given the author an opportunity for some revision and expansion, more especially in those sections devoted to physical signs, and for the addition of the writer's views on a little recognised condition known as hilus tuberculosis in the adult, on which little has yet been written. He very wisely holds that "a diagnosis of phthisis must always be, apart from the discovery of the tubercle bacillus, a matter of probability more or less great: according to the value and number of the various pieces of evidence collected together will the result come near to certainty." The physician has to ask himself in what particular direction does the evidence before him fall short, and then apply those tests which best supply the missing evidence. The book mainly consists of a description of those numerous tests on which a diagnosis of phthisis must rest. The chapter on Hilus Tuberculosis (p. 91) is worthy of careful study. This is the common form of tuberculosis in childhood, when the disease starts in the root glands and creeps outward along the lymphatics surrounding the bronchi and vessels. In the adult the scars of these early disease processes are visible in the X-ray plate, but they occasion no symptoms and give rise to no physical signs. It is only when an exacerbation of disease occurs and gives rise to fresh symptoms and signs that the term hilus tuberculosis becomes admissible and necessary in the adult. The appreciation of the reflex bands of dulness associated with the author's name, and the lung reflex described by Abrams in 1903 (pp. 34–36) requires much training, and must often be matters of uncertainty requiring further evidence to give them value in diagnosis.

**Pye's Surgical Handicraft.** Edited by W. H. Clayton-Greene, B.A., M.B., B.C. Eighth Edition. Pp. xvi., 639. Bristol: John Wright & Sons Ltd. 1919. 21s. net.—In this, the eighth edition of a well-known work, the process of revision and addition has produced a volume of 600 odd pages. There are signs that the present editor, realising that he was on hallowed ground in dealing with such a classic, hesitated to use the blue pencil when well he might. Who, for instance, in the last twenty years ever heard of a patient with strangulated hernia being treated by being immersed in a hot bath until he felt faint, the while taxis was applied? It seems a mistake to devote five pages to an account of the theory and technique of the Wassermann reaction, and only spare one for the treatment of gonorrhœa. It is wrong to say that the object of Janet's method is to *force* fluid into the bladder. It is rather to coax the sphincter to let the fluid pass, with the patient's active co-operation. In order to test the claim that the book has been brought up to date, the reader will turn to the section on war surgery. He will find the subject briefly but adequately treated; but we should have liked to find a reference to a late but most successful method of wound-treatment—delayed primary suture. We are glad to find a warning against haphazard removal of foreign bodies, unless they are causing or threatening trouble. The book contains a vast amount of information of use to those for whom it is written. It is well produced and indexed and, as a whole, illustrated, but the skiagrams are disappointing. It will, no doubt, continue to enjoy the favour it has deserved and received for more than a generation.

**The Forms of Alcoholism and their Treatment.** By Hugh Wingfield, M.A., M.D., B.C. (Cantab.). Pp. 76. London: Oxford Medical Publications. 1919. Price 5s. net.—The treatment of alcoholism is one of the medical practitioner's most difficult problems. The failure of institutional treatment and the disappointing results obtained from the exhibition of drugs are generally agreed upon. In this brief work Dr. Wingfield shows that drug treatment in order to be effective and permanent should be supplemented by treatment by suggestion under light hypnosis. The chapters devoted to the classification of the various forms of alcoholism contain much that is fresh and interesting, and the author has succeeded in producing a work which, though the matter is compressed as much as possible, is full of most valuable help in the treatment of this condition.

**Military Surgery.** By DUNLOP PEARCE PENHALLOW, S.B., M.D., Major, Med. Res. Corps, U.S.A. With Introduction by Sir ALFRED KEOGH, K.C.B. Second Edition. Pp. xx., 555. London: Oxford Medical Publications. 1918. Price 21s. net. —The author of this excellent war manual has been working at military surgery in a large base hospital since the beginning of the war. The rapid exhaustion of the first edition is the best evidence of the appreciation which the book has received. The present edition attempts to give an account of the various theories and practices which have recently been introduced into the service of war surgery. Carrel's method, therefore, is fully described, and there are a number of new illustrations relating especially to the treatment of fractures. Perhaps the main drawback from which the author has suffered is the fact that he has not had the opportunity of dealing with either the earliest or the latest phases of gun-shot injuries, and the book is somewhat lacking both in respect of the casualty clearing station organisation and also with regard to the problems of reconstructive surgery. The illustrations in their number and variety give a good idea of typical war wounds, but as a manual of instruction it would gain greatly by the addition of diagrams and clear teaching about such practical matters as the treatment of fractures. The book is a fine evidence of the author's energy and systematic work during the stress of an active campaign, and it will probably remain as a most useful book of reference.

**The Operative Treatment of Chronic Intestinal Stasis.** By Sir W. ARBUTHNOT LANE, Bart., C.B. Fourth Edition. Pp. xii., 328. London: Oxford Medical Publications. 20s. net.—The previous editions of this book have been widely read. In the present volume little more than one-third is from the pen of the illustrious surgeon whose name appears on the title-page; the remaining chapters are contributed by an anatomist, a pathologist, a bacteriologist, a physician, a skiagraphist, a cardiac specialist, a gynæcologist, an eye-surgeon, a blood specialist, and a dentist, so far-reaching are the effects of the condition claimed to be! However, even if we are not yet convinced that the large intestine and the ileal kink are the root of all evil, it is undoubtedly true that chronic intestinal stasis is a common and important disease, and it is really necessary for every doctor to make himself acquainted with the signs and symptoms and available means of treatment. Only in this book is the subject fully presented, and if read in a properly critical spirit it is of fundamental value. It is possible to admit this much without being a very enthusiastic advocate of the operation called colectomy.

**On Cancer of the Tongue.** The Bradshaw Lecture of 1918. By D'Arcy Power, M.A., M.B. Oxon., F.R.C.S. Pp. 21. Bristol: John Wright & Sons Ltd. 1919.—The Lecture opens with an interesting account of the literary history of cancer of the tongue, from which it appears that the first definite notice of the disease the writer has been able to find is that in some chirurgical lectures to the United Company of Barber Surgeons in 1635—two hundred and eighty-four years ago. Next the zoological distribution of the disease is considered, for it occurs in many vertebrate animals, though in domesticated animals it is very rare, and has not apparently increased in frequency. The subject is next approached from the statistical side. The increase in the death rate from cancer of the tongue is shown to have been great since 1868—the rate has more than trebled in the male sex. Statistics before 1868 are not available. Finally, an estimate of the effect of various possible causative factors is given, concluding a very interesting survey of the subject.

**Back Injuries and their Significance under the Workmen's Compensation and other Acts.** By Archibald McKendrick, F.R.C.S.E. Pp. viii., 173. Edinburgh: E. & S. Livingstone. 1916. Price 2s. 6d. net.—As the title of this little book indicates, it has been designed to assist medical and legal examiners in drawing correct conclusions as to the nature, degree of severity, and prognosis in back injuries, specially of the less severe forms of such injuries, in which there is often much difficulty in "estimating the true value of the subjective symptoms in the comparative absence of physical signs." The mechanical, dynamical, anatomical and physiological considerations bearing on the subject of back injuries are thoroughly considered and instructions given on the best methods of examining and reporting on them for legal or other purposes. The writer's observations on X-ray work in this branch of surgery are particularly valuable, for his experience has been very large. We consider that the book fulfils well the purposes for which it has been written, and will prove helpful to all readers who have to deal with the back injuries of those claiming compensation under various legal enactments.

**Bipp Treatment of War Wounds.** By RUTHERFORD MORISON. Pp. vi., 72. London: Oxford War Primers. 1918. Price 2s. 6d. net.—In this little book the author of this method of treatment gives a full account of the technique, with illustrative cases. He lays stress on the fact that to be successful in the use of it the surgeon needs to be careful rather than clever, and here he probably explains the differences of opinion of those who have made use of it. Many surgeons are enthusiastic advocates, some see little virtue in it and no small danger from poisoning from iodoform or bismuth. The truth, doubtless, lies somewhere between the two extremes, and any surgeon who follows the author's directions with care will certainly find that it can produce remarkable results. Anyone who wishes to make or renew acquaintance with the technique will find all the guidance he requires in this little monograph.

**Burns and their Treatment.** By J. M. H. MACLEOD, M.D., F.R.C.P. Pp. xvi., 160. London: Oxford War Primers. 1918.—One of the last of the Oxford War Primers, and one of the best books on the subject which we have seen. It deals with burns especially from an ætiological point of view, and gives the treatment of the various varieties of burns in detail. The open method of treatment and that by the paraffins are discussed fully. The last chapter is devoted to dermatitis from high explosives, and represents all the most recent work on the subject. The book is well illustrated, and can be recommended to all readers interested in the subject.

**Gymnastic Treatment for Joint and Muscle Disabilities.** By Brevet-Col. H. E. DEANE, R.A.M.C. Pp. 146. London: Oxford Medical Publications. 1918. Price 5s. net.—This manual gives a clear and concise account of the value of a well-equipped gymnasium in the treatment of the disabilities arising from the war. The Indian club, vertical rope, parallel bars, sparred plank, beam and wall bars are clearly described, with exercises suitable in each case. The illustrations are clear. Stress is placed upon the advantage of active muscular exercises compared with the passive movements so commonly employed; also upon the absence of the monotony liable to be associated with elaborate and expensive machines. The book is permeated with the enthusiasm so necessary for successful treatment in this type of disability.

**A Medical Field Service Handbook.** By C. MAX PAGE, M.S., F.R.C.S. Pp. xiv., 159. London: Oxford War Primers. 1919. Price 6s. net.—A vast amount of practical advice has been compressed into a small space in this manual. It is divided into two sections, the first half dealing with general diseases, the second half with wounds. Under the treatment for hemorrhage the statement that intravenous gum solution gives almost as good results as blood transfusion is open to criticism. The chief value of the book is for the medical officer undertaking field service. It is unfortunate that it was not available for use at an early period of the war.

**Surgical Applied Anatomy.** By Sir FREDERICK TREVES, Bart, G.C.V.O., C.B. Seventh Edition. Revised by ARTHUR KEITH, M.D., and W. COLIN MACKENZIE, M.D. Pp. x., 702. London: Cassell & Co. Limited. 1918. Price 10s. 6d. net.—We are very pleased to welcome another edition of this valuable manual, which has been thoroughly revised and again brought up to date. A further addition of sixteen coloured illustrations and considerable additions in "orthopædic anatomy" enhance the value of the book. Important points are more clearly emphasised in the text by a much freer use of heavy type in the printing. The new anatomical nomenclature has been added in brackets side by side with the old.

**National Health: from Magic, Mystery, and Medicine to a National Health Service.** By FERDINAND REES, M.D. Pp. 68. Bristol: John Wright & Sons Ltd. 1919. Price 1s. 6d. net.—This is an interesting discussion of some of the problems in the reorganisation of medicine at the present time. The writer is a warm advocate of a national health service on the lines of an improved national health insurance. He is deeply impressed with the evil effects of competition for practice amongst doctors, and thinks that higher aims and greater efficiency in the struggle against disease could be obtained in a well-organised public service. Though he seems to minimise the immense advance in public spirit and practice actually realised during the individualist system of private practice, and to regard the voluntary hospitals as entirely out of date, his criticisms are suggestive and often very valuable. The book is eminently readable, but the problems are too complex to be fairly discussed in the sixty-eight pages he devotes to them.

**The Indian Operation of Couching for Cataract.** By ROBERT HENRY ELLIOT, M.D., B.S. Pp. xii., 94. London: H. K. Lewis & Co. Ltd. 1917. Price 7s. 6d. net.—We have a threefold addition to medicine in this volume. First, a fascinating bit of history of the ancient and modern Oriental custom of operating for cataract by depressing the lens, incidentally giving a vivid picture of the ignorant and superstitious Eastern mind. At the same time as much credit as possible is given to the operators for doing what they truly believe to be the best for their patients. Second, a painstaking monograph on the pathology and results of this operation in a series of over 750 cases, including 54 excised globes, showing in excellent plates the various conditions which have followed this crude and septic operation. Twenty-five years had been spent in collecting this material. Statistics show carefully and fairly that scarcely 10 per cent. have better vision than 6/18. 30 per cent. show iritis and irido-cyclitis, and another 10 per cent. consecutive glaucoma. Third, prevention. The appalling results as compared with extraction cannot fail to influence those responsible for the health of the natives, and will assuredly bear fruit in combating superstition, and thus saving the sight of thousands a year in a large portion of our empire.

**Practical Vaccine Treatment for the General Practitioner.** By R. W. ALLEN, M.A., M.D. Pp. xii., 308. London: H. K. Lewis & Co. 1919. Price 10s. 6d.—One feels in reading this book that it contains a special pleading—in places almost more than special—and yet as the arguments are unfolded and the experiences are aggregated there comes an understanding of its necessity. Vaccine therapy has been over-boomed, misapplied, cast aside, all without rhyme or reason. The temperamentally conservative shunned it or abused it; the opposite nature used it without discretion—perhaps without sufficient knowledge. Manufacturers hailed it as a godsend for their balance sheets; retailers dispensed its medicaments over the counter. The bacteriologist treated it with the utmost caution, shuddering often at the attempts of amateur vaccinists and wondering about the possibilities which might befall the vaccine or the patient. So runs the script for practitioner, consultant and pathologist. Plain fare for all. Collect specimens properly. If you cannot, call in a bacteriologist, or, preferably, a vaccine therapist. Make opportunities for bringing the pathologist to the patient. Watch your patient day by day after each injection. Associate the course of injection with thought and observation, not with rules of thumb. Shun stock vaccines unless it is impossible to obtain others. Differentiate between sera and vaccines. These are some of the impressions left after perusal. Yet the dogmatism, insistence and personality of the book will be forgiven when it is realised how hard is the fight for efficiency and scientific accuracy. The author hopes that he will be accorded the right to be included within the category of the last line of an old Persian proverb, which says, "He who knows and knows that he knows is a wise man, follow him." His pathway takes us through a chapter on toxins and antibodies, along the narrow track leading to the collection of material and the preparation of vaccines, down the broad road to the rocky whirlpool of prophylactics and therapeutics. All the systemic diseases are treated in turn, and the volume closes with a series of questions from practitioners and answers by the expert. There is a good index. The book is for all to read and re-read, partly as an exercise in separating wheat from chaff, or again, as stimulus to thought, contentious or otherwise, and finally as a chastisement with scorpions for our sins of omission or commission.

**A Manual of Elementary Zoology.** By L. A. Borradaile, M.A. Second Edition. Pp. xii., 616. London: Henry Frowde and Hodder & Stoughton. 1918. Price 16s. net.—This new edition presents various difficulties which in our opinion could easily have been avoided. If the book is an elementary text-book especially for medical students, the author ought not to assume any knowledge of physics and chemistry, and consequently ought to treat more fully such questions as respiration, digestion, excretion and the physical laws determining division, growth and surface area in relation to cubical content. Again, it would lead to a much better conception of zoology if one could advance from simple to complex along reasonably natural lines, and the subject-matter were rearranged, the systematic part commencing with Amœba. One or two minor faults creep in, while a few of the old favourites still retain their place. Why is it necessary to refer to the internal opening of the vas deferens in the earthworm as the "sperm rosette"? To us this seems just an unnecessary addition to terms in a subject already overburdened with them. There is a curious misstatement in reference to *Filaria bancrofti* on page 304, and a slip has occurred in the diagram of the cranial nerves of the skate. We are not sure that the long appendix on practical work is really needed, and even if so the diagram of a compound microscope might have been replaced by that of a more modern type of instrument. The book is, however, well written and illustrated with a large number of excellent diagrams.

**Diseases of the Throat, Nose and Ear.** By W. G. Porter, M.B., B.Sc., F.R.C.S. Ed. Third Edition. Revised and edited by A. Logan Turner, M.D., F.R.C.S. Ed. Pp. xv., 30. Illustrated. Bristol: John Wright & Sons Ltd. Price 12s. 6d. net.—We extend a warm welcome to the third edition of this very practical, sound and scientific epitome of the essentials of diseases of the throat, nose and ear, which so well fulfils the main object of the writer, to provide the practitioner and senior student with a moderate-sized hand-book. The earlier editions were deservedly well received. This, the third, has been improved by the revision of the section on vestibular tests, the amplification of the section on nerve deafness and otosclerosis by Dr. J. S. Fraser, and other contributions by Dr. Douglas Guthrie and Dr. Gardiner. These colleagues have joined with Dr. Logan Turner in revising the text-book of the author, whose death in action during the war

FIG. 12, p. 45.

is a grave loss to British oto-laryngology. The illustrations are very good and numerous, and the publishers may be congratulated on the general excellence of the printing and production of the work. By the courtesy of the publishers we are enabled to reproduce one of the illustrations.

**An Introduction to General Physiology, with Practical Exercises.** By W. M. BAYLISS, M.A., D.Sc., F.R.S. Pp. viii., 238. London: Longmans, Green & Co. 1919. Price 7s. 6d. net.—*The Principles of General Physiology*, published in 1914 by Professor Bayliss, beyond question marks an epoch in British physiology. We must go back to 1876, the year of publication of *Foster's Text-book of Physiology*, to find a parallel to the interest aroused by the appearance of Bayliss's work.

The present volume of about 240 pages is essentially an elementary exposition of the subject on the lines of his larger treatise. Whatever may have been the opinions held as to the part which physical chemistry was to play in the investigation of physiological processes, it is now certain, as a perusal of this book shows, that marked progress has been accomplished by the application of the methods of physical chemistry to physiological studies. The publication of this book is in itself a proof of this statement. The treatment of the subject is clear; that it is somewhat dogmatic cannot be held as a fault. The requirements of no particular examination have been taken into consideration and in this lies much of the scientific excellence of the book. It is not burdened with many illustrations, for Professor Bayliss holds, and in this the majority of teachers will agree, that a picture which attempts to represent what a part of a living organism actually looks like is less instructive than a diagram which frankly attempts no more than to indicate what is essential to the working of the mechanism. In Fig. 4, p. 109, a diagram of the possible mechanism of the organ of Corti may be noticed, also the methods for measuring the degree of electrolytic dissociation on pages 173–175. The whole book,—Part I., The Text, and Part II., Laboratory Work—is original in conception, and may be cordially recommended for perusal, not only by students, for whom it has primarily been written, but by all interested in the modern trend of physiology.

**War Wounds of the Lung.** By PIERRE DUVAL. Authorised English Translation. Bristol: John Wright & Sons Ltd. 1918. Pp. viii., 99. Price 8s. 6d. net.—In no province of treatment have our ideas been more completely revolutionised by the experience gained in the war than in that which concerns gunshot injuries of the lungs and pleura, and to no one more than to Pierre Duval has this startling change been due. The volume of which the present work is an excellent and painstaking translation was originally published in 1917, but before its appearance Duval's methods and opinions had gained considerable notoriety among British medical officers in France, and much new and valuable work had been carried out by them on lines similar to his, if not always identical. The principle of non-interference in wounds of the chest was a tradition from the South African War, and was "official" at the beginning of the Great War in 1914. The development, however, of well-equipped operating stations close behind the firing-line, and the enthusiasm and skill of good surgeons, rendered possible the innovations which find their expression in this book. The two great dangers which threaten a man wounded in the chest are early death from collapse or hemorrhage, and, later, the occurrence of severe and hardly less fatal septic infection. These two dangers it has been the work of the surgeons to prevent, by early treatment of an operative character. The earlier dangers have been met by operations for arresting hemorrhage at its source and for closing the thorax where a large open wound had produced collapse of the lung, and its attendant shock with embarrassment of respiration. The later danger, that of sepsis, has been met by the operation of thoracotomy, done as soon as circumstances permitted, with a complete toilet of the pleura, removal of damaged tissues and in many cases of shell fragments embedded in the lung itself.

The results cannot really be adequately appreciated by statistics, although Duval gives some striking figures. The success of the modern methods depends so much on the circumstances of warfare and the skill and experience of the medical officers concerned, that the only real evidence of their value is that afforded by those who have actually watched them and assisted in carrying them out.

This book is, for all its modest proportions, a classic of the war; its English dress suits it admirably; and we commend it to all who are interested in the progress of surgery as a model of what a monograph dealing with original work should be. It will appeal, moreover, not only to surgeons but to physicians, for the subject is one in which both are equally concerned. The best results have always been obtained by close collaboration between the two branches of the healing art, a fact which is eloquently expressed in the author's dedication to his colleagues of the Auto-Chir, No. 21.

**Surgical Operations: A Text-book for Nurses.** By E. W. Hey Groves, M.D., M.S., F.R.C.S. Pp. viii., 255. London: Oxford Medical Publications. 1919. Price 21s. net.—The author of this volume has already won universal renown among medical students of all ranks by his *Synopsis of Surgery*, and the same success is certain for this companion work for the nursing profession whose desire is to understand the processes of operations and the why and wherefore of the surgeon's needs.

The book opens with a chapter describing the most up-to-date aseptic and antiseptic preparations for surgical operations, and is a fine, clear appeal to the surgical conscience. There follows a series of chapters outlining all the common operations, both minor and major, which is remarkable for its concise context, and the inclusion of the tested advances that remain out of the vast research in wound surgery that the recent war has afforded. But undoubtedly the supreme merit of this work lies in the profusion of most animated and illuminating sketches of the various fields of operation to which the text alludes.

This very up-to-date addition to medical literature concludes with an illustrated catalogue of the essential instruments of surgical handicraft, which is a genuine boon, and a fitting close to a picturesque as well as scientific survey of operative surgery.

The clearness of the type and illustrations are a great tribute to the publishers.

**Encyclopædia Medica.** Second Edition. Vol. vi. Pp. viii., 658. Edinburgh: Wm. Green & Son. 1919.—Another volume of this great work is on similar lines both as regards author, revisers and subjects. Thirty-four authors write special articles on subjects ranging from H to I under the general editorship of Dr. J. W. Ballantyne, who is himself the author of one of these. An article of one hundred pages on Insanity is included by the late much-lamented Dr. C. A. Mercier, whose voluminous writings on Psychology and kindred topics will long be remembered. In commenting on the treatment of insanity he emphasises the necessity for the reduction of function of the supreme regions of the nervous system, and he adds the remark that "if every insane person is to receive what is humorously termed hospital treatment—that is to say, is to be kept in bed—the treatment is not consistent unless he is also relieved by a catheter and fed with a stomach-pump." It is greatly to be regretted that we shall have no further output from Dr. Mercier's active brain and facile pen.

**A Guide to Gynæcology in General Practice.** By COMYNS BERKELEY, M.A., M.D., M.C., and VICTOR BONNEY, M.S., M.D. Second Edition. Pp. xxiii., 467. London: Oxford Medical Publications. 1919. Price 31s. 6d. net.—This book has been written expressly for the general practitioner, and no operative procedures or pathological details are included in the subject-matter, the general arrangement of which is somewhat unusual.

Part I. is devoted to a description of the various methods of abdominal and vaginal examination. In it the authors advocate the semi-supine as the position of election for bimanual vaginal examinations. It certainly has the advantage that it entails less exposure of the patient, but inasmuch as it does not procure such complete relaxation of the abdominal muscles as the lithotomy position, it can hardly be considered so satisfactory.

Part II. deals with the significance of symptoms at considerable length, the section on abdomino-pelvic pain being particularly useful.

Part III. is occupied with the interpretation of physical signs. This is dealt with in an exhaustive and comprehensive manner, and should be of considerable assistance in the diagnosis of obscure pelvic lesions.

Part IV. describes the various methods of treatment, including those by radium and X-rays. In it the authors express themselves as somewhat adverse to the use of the pessary. There is, however, no doubt that these instruments still have a fairly wide field of usefulness, more especially in general practice, many women still being unwilling to submit to operation if their symptoms can be relieved by wearing a pessary. The authors lay much stress, and rightly so, on the importance of early exploration of the uterus in cases of irregular or continuous uterine hemorrhage.

The fifth, and last, part contains a concise account of the medico-legal aspects of gynæcology. This should enhance the value of the book. The text is illustrated by a number of clear and useful figures, and the book ought to be of great assistance to the general practitioner in dealing with the gynæcological cases which he meets in his practice.

## Figures and Tables

**Fig. 12 f1:**